# An Automatic Bleeding-Rank System for Transurethral Resection of the Prostate Surgery Videos Using Machine Learning

**DOI:** 10.3390/diagnostics11101767

**Published:** 2021-09-26

**Authors:** Jian-Wen Chen, Wan-Ju Lin, Chun-Yuan Lin, Che-Lun Hung, Chen-Pang Hou, Chuan-Yi Tang

**Affiliations:** 1 Department of Computer Science, National Tsing Hua University, Hsinchu 30013, Taiwan; vito9580work@gmail.com (J.-W.C.); cytang@cs.nthu.edu.tw (C.-Y.T.); 2 Department of Mechanical Engineering, National Taiwan University, Taipei 10617, Taiwan; d05522001@gmail.com; 3 Department of Computer Science and Information Engineering, Asia University, Taichung 41354, Taiwan; cyulin@asia.edu.tw; 4 Institute of Biomedical Informatics, National Yang Ming Chiao Tung University, Taipei 11221, Taiwan; 5 Department of Computer Science and Communication Engineering, Providence University, Taichung 43301, Taiwan; 6 Department of Urology, Linkou Chang Gung Memorial Hospital, Taoyuan 33302, Taiwan; 7 School of Medicine, Chang Gung University, Taoyuan 33302, Taiwan; 8 Graduate Institute of Clinical Medical Sciences, College of Medicine, Chang Gung University, Taoyuan 33302, Taiwan; 9 Department of Computer Science and Information Engineering, Providence University, Taichung 43301, Taiwan

**Keywords:** ranking of bleeding level classification, ResUnet model, transurethral resection of the prostate (TURP)

## Abstract

Benign prostatic hyperplasia (BPH) is the main cause of lower urinary tract symptoms (LUTS) in aging males. Transurethral resection of the prostate (TURP) surgery is performed by a cystoscope passing through the urethra and scraping off the prostrate piece by piece through a cutting loop. Although TURP is a minimally invasive procedure, bleeding is still the most common complication. Therefore, the evaluation, monitoring, and prevention of interop bleeding during TURP are very important issues. The main idea of this study is to rank bleeding levels during TURP surgery from videos. Generally, to judge bleeding level by human eyes from surgery videos is a difficult task, which requires sufficient experienced urologists. In this study, machine learning-based ranking algorithms are proposed to efficiently evaluate the ranking of blood levels. Based on the visual clarity of the surgical field, the four ranking of blood levels, including score 0: excellent; score 1: acceptable; score 2: slightly bad; and 3: bad, were identified by urologists who have sufficient experience in TURP surgery. The results of extensive experiments show that the revised accuracy can achieve 90, 89, 90, and 91%, respectively. Particularly, the results reveal that the proposed methods were capable of classifying the ranking of bleeding level accurately and efficiently reducing the burden of urologists.

## 1. Introduction

Benign prostatic hyperplasia (BPH), affecting approximately 210 million men in the word, is the main cause of lower urinary tract symptoms (LUTS) in aging males [1]. Reduced urinary flow and the progression of voiding and storage symptoms are all symptoms of untreated BPH, which can lead to acute or chronic urinary retention (UR) [2]. The sequelae of BPH include decreased urinary flow and progression of voiding and storage symptoms, eventually resulting in acute or chronic urinary retention (UR). Although alpha-1 blockers are used for first-line treatment of BPH in men with LUTS, surgical intervention is an appropriate treatment for patients with moderate-to-severe LUTSs and for patients who have developed acute UR or other BPH-related complications, according to the updated guidelines [3]. Meanwhile, TURP remains the most common and effective treatment for patients who have had a poor response to medication [4]. TURP can also prevent the need for indwelling or intermittent catheterization in the future [5,6]. Despite the fact that TURP is a minimally invasive procedure, bleeding is still the most common complication [7]. Transfusion rates after TURP have been reported as high as 2.9% in a recent multi-institutional study [8]. Although mass bleeding during TURP is uncommon, intraoperative bleeding can obscure surgical vision, resulting in prolonged operative time, capsular perforation, fluid absorption, and the overuse of irrigation fluids, all of which are risk factors for TURP syndrome and sepsis [9]. Therefore, the evaluation, monitoring, and prevention of interop bleeding during TURP are very important issues. There are currently some studies aimed at the evaluation of bleeding during TURP surgery [10], but these methods have to be operated in the laboratory and cannot be monitored in time. We have published a study on the use of artificial intelligence to evaluate bleeding during TURP and proved it feasible and promising [11]. The purpose of this research is to do further analysis on the basis of our research results. We handed the TURP surgical videos to experienced urologists and artificial intelligence to evaluate the severity of interop bleeding and compare the relevance of the scoring results between the two groups.

Recently, deep learning technology has progressed rapidly in the medical field, which mainly focuses on the tasks of segmentation and classification to assist doctors in diagnosing diseases more accurately and rapidly. The demand for the segmentation technique plays an important role in the medical field. Segmenting the detailed features in the complex background is the current requirement of analyzing the medical image. Several researchers employed the semantic segmentation network to detect the complex features of the lesion, tumors, skin lesion, etc. in the medical image. Xu et al. [12] proposed the D-ResUNet network, which combines the structure of ResNet and U-Net to segment the regions of colonoscopy lesions. The proposed network could improve the prediction of the shape and edge contour of cell morphologic information. Peng et al. [13] proposed an end-to-end cascaded deep ResUNet network to segment the liver lesion, which could increase the prediction results of accuracy and sensitivity. Zhang et al. [14] employed U-Net model to segment breast tumors in Dynamic Contrast-Enhanced MRI of 2D and 3D images. Yang et al. [15] proposed a multi-task DCNN technique to segment and classify skin lesions. Due to the high accuracy and efficiency of the segmentation method that this study utilized, the ResUNet model by generating the segmentation masks to eliminate the red light of the cutting loop.

Moreover, classification is also another important method in the application of medical images. In recent years, deep learning techniques have brought a great breakthrough of the classification topic in the medical image. It can be attributed to the characteristic of automatically learning the features in the images. Although deep learning models have the ability to recognize representative features from a large scale of datasets, the small amount of datasets leading to poor recognition results of deep learning models poses a challenge in the task of classification. Due to the small amount of data used in this study for analysis, it is difficult to obtain higher accuracy of the deep learning models, which makes them unsuited for medical application. Machine learning techniques have superior recognition results to overcome the issues of a small number of datasets. Machine learning techniques, such as Bayesian, SVM, KNN, Random forest, have been successful applied in the studies of medical applications. Fisher et al. [16] used the Bayesian network to classify the breast lesions into different pathological categories. Andrés et al. [17] determined whether the patient had the disease of age-related macular degeneration or not by using the method of SVM network and digital image processing. Ramteke et al. [18] adopted the KNN classifier to classify the medical images into normal and abnormal categories, and the proposed method could successfully test the real CT scan brain images. Ko et al. [19] integrated segmentation and random forest model to classify the cell of the nucleus and cytoplasm into five categories. They also proved that the random forest network has superior performance on small training datasets. According to the above studies, the high performance using machine learning in medical images was shown, so this study adopted the machine learning approaches to solve the problem of classifying the level of blood loss. By using a step of segmentation technique in contouring the mask on the cutting loop, and integrating the machine learning approaches of classifying different level of blood loss, which can meet the requirement of the doctors in diagnosing the quality during the operation.

This study aims to assess the status of blood loss during TURP surgery. We proposed a method to extract features of the blood loos from TURP videos by using image segmentation technology. The proposed method consists of three steps: the first step is to remove the area of cutting loop; the second step is to extract the area with red color; the final step is to classify the level of blood loss. Due to the cutting loop producing a red light during surgery, which is similar to the color of blood, the accuracy of the level of the blood loss is decreased. To reduce this affect, the area of the cutting loop is removed from videos by using a semantic segmentation model. The red color region is extracted by YCbCr color space method. Finally, the machine learning technology is used to classify the level of the blood loss. Experimental results show that the cutting loop can efficiently be removed. The accuracy for classifying four ranking of blood levels can achieve 90, 89, 90, and 91%, respectively. By using the proposed method, it can help urologists to analyze the level of blood loss.

This paper is organized as follows: Section 2 introduces the methods for segmentation model and four classification models. Section 3 contains the description of dataset and detailed experimental results with a comparison of the four classical models discussion. Section 4 concludes this article.

## 2. Materials and Methods

The aim of this study was to evaluate the ranking of bleeding levels for TURP surgery. Thus, state-of-the-art classification models are involved. Continuous bladder irrigation was used throughout the surgery to ensure a clear view of the surgical field. Packets of 2000 cc normal saline for irrigation were placed at a height of 100 cm above the patient and the circulating nurses were responsible for ensuring a continuous flow during the whole procedure to maintain the flushing rate at least 150 dpm (drops per min.). The TURP surgery is performed by a cystoscope passing through the urethra and scraping off the prostrate piece by piece through a cutting loop. Moreover, it is easily disturbed to judge bleeding areas for experienced physicians because a cutting loop with red light yielding from the surgical cutting loop often appears on the images. Whereas it is difficult to judge the color space between the blood region and the cutting loop even when an automatic computer-aided technique is utilized. It implies that cutting loop with red light elimination is needed. To solve this problem, the segmentation model was used to eliminate the cutting loop before the level of bleeding classification stage.

**Figure 1:**
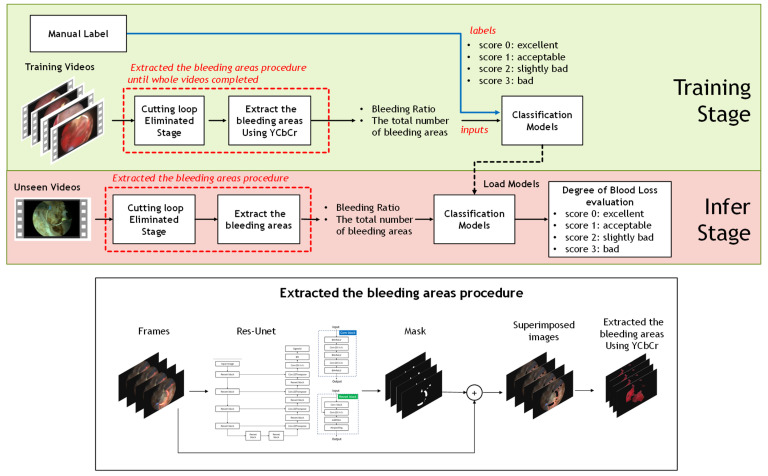
The illustration of the flowchart for ranking the bleeding loss level.

In this section, an overview of the procedures is described as follows. First, approximately 500 pcs surgery frames were collected from each video. Next step, the cutting loop in the surgery frames was removed through the segmentation model. Furthermore, the bleeding ratio and the total number of the bleeding regions, extracted through YCbCr color space, would be estimated until whole the frames were completed for each surgery video. Finally, the popular classification models, e.g., SVM, KNN, Random Forest, and Naive Bayes, were used to infer the level of blood loss. The ground truths of four levels were guided by sufficiently experienced urologists. Finally, the procedure was finished until all TURP videos were completed. The pipeline proposed is shown in Figure 1.

### 2.1. Using Segmentation Model to Eliminate the Cutting Loop

**Figure 2:**
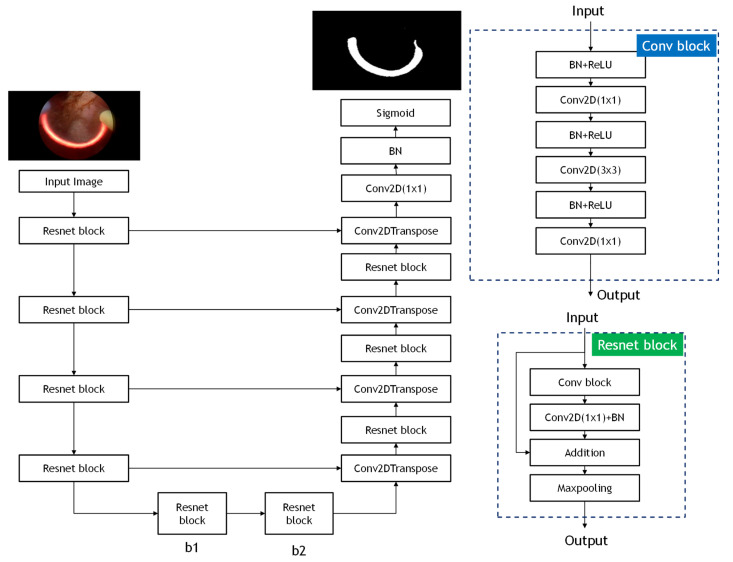
The structure of the ResUnet model.

In this study, the architecture of ResUnet model is used to eliminate the pattern of the cutting loop affecting the accuracy of the classification model. ResUnet model is modified from U-Net model [20], thereby achieving higher performance than the U-Net model. The ResUnet model exploits and integrates the U-Net structure and deep residual learning to the end-to-end model. The structure of ResUNet model consists of three elements, which are encoder, decoder, and bridge. In the encoder part, input images are used to compress the compact representation. The decoder is used to decompress the representation to the pixel-wise classification. The function of a bridge is served as the connection of integrating the encoder and decoder. All of these three elements adopt the method of residual learning, instead of pooling operation, making the complex deep network structure able to be trained more easily. The structure of the ResUnet model is shown in Figure 2.

### 2.2. Machine Learning Classification Models Overview

In this section, given the total number of bleeding areas and bleeding ratio, what was collected from the segmentation stage was used as the classification model input. To assess the ranking of bleeding loss level, the ground truth of four grades, including score 0, score1, score 2, and score 3, were manually label by sufficiently experienced urologists. The popular classification models, e.g., SVM, KNN, Random Forest, and Naive Bayes, were utilized. Specific illustrations are described as follows.

#### 2.2.1. Random Forest

Random forest is one of the most used machine learning algorithms [21]. Random forest can effectively process small amounts of data, so many researches use random forest to deal with classification problems. The random forest algorithm is the extension of the decision tree, which establishes the forest in a random method. There is no correlation between each decision tree, and these trees are used to vote to determine the prediction results. For the classification problem, each tree would provide its own classification choice in the random forest algorithm. The overall output of the random forest is the result of the most voted prediction.

#### 2.2.2. SVM

SVM is a popular supervised learning algorithm of the classifier [22]. Due to it having greater generalization performance than SVM, it has drawn much attention for classification applications. The main purpose of the SVM algorithm is to find the hyperplane that maximizes the margins to separate the categories perfectly. Before finding the hyperplane, the input data should be mapping from low-dimensional space to high-dimensional space. To maximize the margin of the hyperplane, the optimal separation of the hyperplane is shown as Equations (1) and (2).

(1) $$\min\emptyset \left(w\right)=\frac{1}{2}{\left\|w\right\|}^{2}+C\left[\overset{N}{\underset{i=1}{\sum }}\delta_{i}\right]$$

(2) $$g\left(x\right)=sgn\;\left[\overset{m}{\underset{i=1}{\sum }}\alpha_{i}^{*}y_{i}K\left(x_{i}\cdot x\right)+b^{*}\right]$$

where the pair of (*w*,*b*) is defined as the separating hyperplane. *C* is the regularization parameter. $\delta_{i}$ is defined as the slack variable. For the nonlinear hyperplane, the decision function is given as Equation (2). Where *K* kernel satisfies the Mercer condition. (*x_i_*,*y_i_*) are the training samples. The support vector is defined as$\;\alpha_{i}$.

#### 2.2.3. K-Nearest Neighbor (KNN)

KNN is classic supervised learning of machine learning [23]. Because KNN model does not have a training stage, it is also called the lazy learning algorithm and has been widely applied in real applications. The core of the KNN algorithm is to calculate the distance between the training dataset and the testing sample and choose the nearest neighbors of the data points to discriminate the category of the target point. In this study, Euclidean distance is used as the distance function, which is shown as Equation (3).

(3) $$\mathrm{dist}\;\left(A,B\right)=\sqrt{\frac{\sum_{i=1}^{m}{\left(x_{i}-y_{i}\right)}^{2}}{m}}$$

where *A* represented the features vectors of (*x*_1_, *x*_2_, …, *x_m_*), B represented the feature vectors of (*y*_1_, *y*_2_, …, *y_m_*). m is the dimensionality of the feature space.

#### 2.2.4. Naïve Bayes

The Naïve Bayes algorithm has been proven an effective method to deal with medical diagnosis and text classification [24]. Naïve Bayes is a classification method based on probability and statistics, which calculates the probability value from a given dataset. The algorithm utilized the concept of Bayes with the condition of independence hypothesis, which can solve the task of high-dimensional data.

## 3. Experiment and Results

In this section, the classification and segmentation of the deep learning models proposed in this study are described and investigated in detail. These models were trained in the Graphics Processing Unit (GPU) embedded with NVIDIA GeForce GTX 1080 Ti for computational acceleration. The deep learning framework Keras was used together with TensorFlow, a machine learning backend library.

### 3.1. Dataset Description

We randomly selected and edited 287 surgical video clips (by ADOBE PREMIERE PRO CC 2019 v.13.1.5.47 WIN/MAC) from the complete recording videos of 50 different TURP procedures. Each clip lasted 3 min. The surgical procedures were performed by a single surgeon, using the Olympus SurgMasterUES-40 bipolar generator and the OES-Pro bipolar resectoscope (Olympus Europe, Hamburg, Germany). The standard settings of energy were 200 and 120 W for cutting and coagulation, respectively. The edited videos were handed over to three urologists of Chang-Gung Memorial Hospital, Linko, Taiwan, to evaluate the level of interop blood loss of each video clip. The evaluators all have sufficient experience in TURP surgery, and experience in performing this procedure were 7 years, 9 years, and 15 years, respectively. The three evaluators independently scored each of the 287 surgical video clips based on the visual clarity of the surgical field (score 0: excellent; score 1: acceptable; score 2: slightly bad; and 3: bad). If the scores given by the 3 evaluators were exactly identical, the score obtained is the final score for this video clip. If two of the evaluators gave the same score while the other gave differently, the score given by the two evaluators was admitted. If the scores given by 3 evaluators were completely different, this video clip was taken out for re-evaluation and scored again.

### 3.2. Evaluating for the Segmentation Model

In an attempt to reduce the effect of the cutting loop with red light, the process of segmentation is a major task in this study. In the training stage, approximately 80 images were selected as a training dataset for the segmentation model. To evaluate the performance of the segmentation model, around 30 unseen images with a red light pattern, randomly collecting from different videos, were taken as a testing dataset. There are a few testing samples shown in Figure 4. Figure 4a,b present the input image for testing and the bleeding areas were extracted through YCrCb color space. Figure 4c,d indicate the regions of the cutting loop were eliminated via segmentation model and the total numbers of the bleeding area were revised. The result illustrates that the total number of the bleeding area was significantly calibrated while cutting loop regions were eliminated, thereby the correct ranking of bleeding level can be effectively classified properly. Apart from this, the performance of the ResUnet model is assessed with the indicator of the intersection over union (IoU) and Dice coefficient (DC). The indicator of IoU and DC are the standard indexes to evaluate the performance of the segmentation model. Both of these two indicators were compared with the correct answer of ground truth. The definition of IoU and DC is given in Equations (4) and (5):

(4) $$\mathrm{IoU}=\frac{Predict\;\cap^{\;}\;GT}{Predict\;\cup^{\;}\;GT}$$

(5) $$\mathrm{DC}=2\times \frac{\left|Predict\;\cap^{\;}\;GT\right|}{\left|Predict\right|+\left|GT\right|}$$

**Figure 3:**
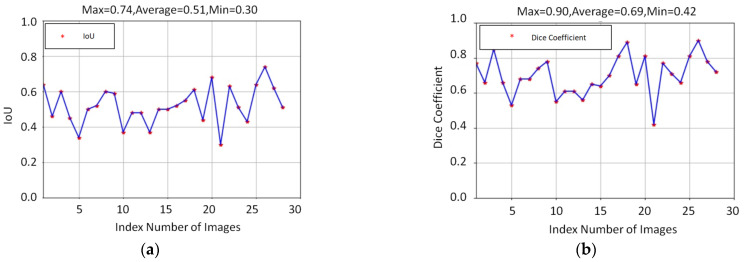
The IoU and Dice Coefficient for ResUnet model.

**Figure 4:**
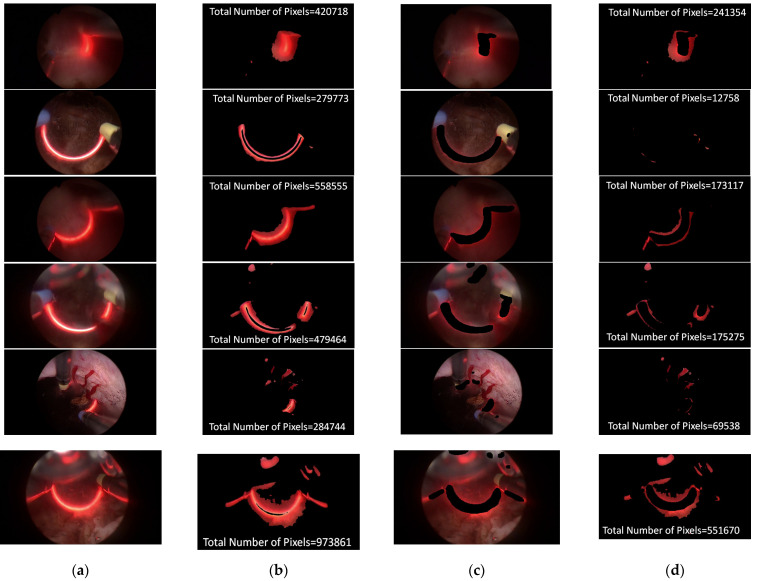
(**a**) Input image. (**b**) The bleeding areas of input image were extracted through YCbCr. (**c**) The image with cutting loop eliminated. (**d**) The image with revised bleeding areas.

Both IoU and DC are used to measure the similarity between the predicted area and the ground truth of the segmentation model results. The value of IoU is the ratio of the intersection and the union for the predicted area and the ground truth. The range of the IoU value is from 0 to 1. The value 0 represents no overlap and the value 1 indicates the identicalness between the region of prediction area and ground truth. The Dice coefficient is defined as two times the region of overlap divided by the sum of the predicted area and the ground truth. The meaning of DC is the same as the IoU value, if the DC value is 1, the output of segmentation achieves the best result. The results of IoU and Dice coefficient for ResUnet model is given as Figure 3. The average IoU and Dice coefficient of ResUnet model are 0.51 and 0.69, respectively. Furthermore, the prediction results of the ResUNet model are shown in Figure 4, where ResUNet model could mark the region of red light pattern efficiently. This study also compared the results of the bleeding area, which adopts the ResUNet model to evaluate whether the red light pattern can be removed or not. This is because the color of red light is similar to the bleeding that the red light can greatly affect the level of distinguishing the bleeding area. According to Figure 4 of the bleeding area, the bleeding area by using ResUNet model can reduce misjudgment so that the red light disappeared in the bleeding area.

### 3.3. Performance of the Classification Models

To evaluate the ranking of bleeding level, the four state-of-art machine learning classification models, e.g., SVM, KNN, Random Forest, and Naive Bayes, were implemented in this study. Each ground truth of surgical video was manual label into four grades, including score 0: excellent, score 1: acceptable, score 2: slightly bad, and 3: bad. In the training stage, approximately 150 videos were selected as a training dataset for classification models. Apart from this, 10% of the training dataset was divided into validation datasets and the other datasets were used for training dataset. Furthermore, around 137 surgical videos were used to evaluate the performance of classification models in the testing stage.

#### 3.3.1. Scatter Plot of Classification Model Results

To grasp the prediction for each surgical video more clearly, the classification results were visualized. To this end, a qualitative comparison of the four machine learning models is given to visualize the prediction results of the classifier. More details of the classifier results are provided in the following section. The scatter plot in the validation stage and testing stage are shown in Table 1 and Table 2. Given total numbers of blending areas and bleeding ratio, collecting from the segmentation stage, were used as the input of classification model. The bleeding ratio was taken along the y-axis, and the total number of the bleeding regions was taken along the x-axis, where the unit represented the normalization of percentages and pixels, respectively. Each circle pattern indicated the difference of prostate surgical video. The four colors used to represent four types ranking of blood levels, where yellow, green, blue, and purple illustrated score 0, score 1, score 2, and score 3, respectively. The four different decision boundaries were generated through the different classification models in the training stage. In the validation stage, around 11 videos were classified and placed in the corresponding decision location. Consequently, the accuracy obtained was 0.55, 0.64, 0.73, and 0.64 via KNN, Naive Bayes, Random Forest, and SVM, respectively. To analyze the classifier effect on the blood loss dataset, the confusion matrix is used to visualize the performance of an algorithm. The confusion matrix is one of the most commonly utilized methods in the supervised classification task. It provides more detailed analysis to understand the predictive ability of the classifiers for each category. The confusion matrix not only can examine the errors being made by the classifier, but also understand the types of errors of the classifier being discriminated. In the confusion matrix, the meaning of each column represents the predicted categories, and each row represents the actual categories. The results reveal that the Random Forest classifier achieves the most outstanding predictive ability for each category. In addition, many indicators also can be extended to understand the performance of the classifier in different aspects through the confusion matrix.

For the prostate surgery prediction, the error would not significantly affect the result for the urologists so that the slight error was allowable in this work. The prediction of surgery video was acceptable if it would not be predicted as cross-level. The revised confusion matrix is shown in Table 2. For example, the point, which was predicted as score 0 or score 2, can be allowed if it belongs to score 1. Thus, the accuracy can be revised as 90, 89, 90, and 91% respectively in a testing stage.

#### 3.3.2. Quantitative Evaluating of Classification Model

To quantify the performance classification model, the popular used indicators are accuracy, precision, and recall. The definition of accuracy, precision, and recall are shown in Equations (6)–(8), where the accuracy means the ratio of correctly classified blood loss among all predicted categories. The precision refers to the corrective proportion among the total number of predicted blood loss images, and recall represents the incorrect proportion of classified blood loss images in each category. In Equations (6)–(8), true positive (*TP*) is the blood loss images correctly classified for the classifier; true negative (*TN*) is number of the blood loss images not correctly classified for the classifier; false positive (*FP*) is the number of non-correctly classified of the blood loss images to the correct images; false negative (*FN*) is the number of non-correctly classified blood loss images to the non-correct images. The comparison of the four machine learning classifiers considering before and after the revised process is given in Table 3.

(6) $$Accuracy=\frac{TP+TN}{TP+TN+FP+FN}$$

(7) $$Precision=\frac{TP}{TP+FP}$$

(8) $$Recall=\frac{TP}{TP+FN}$$

### 3.4. Correlation Coefficient between Ground Truth and Predictions

To demonstrate the elimination of cutting loop is a major task for TURP, the comparison of classification results between the cutting loop pattern reserving or not is shown in Table 4. In contrast to other classification models, the performance of the Naïve Bayes model is the most outstanding among all the models. Therefore, the Naive Bayes model is selected as the classification model. Apart from this, the correlation coefficient is utilized to evaluate the ranking of bleeding level relationship between ground truth and two system predictions. The correlation coefficient is used to measure how strong a relationship is between two variables. The correlation function is shown as Equation (9):

(9) $$Correlation\;Coefficient=\frac{\sum_{i=1}^{n}\left(x_{i}-\mu_{x}\right)\left(y_{i}-\mu_{y}\right)}{\sqrt{\sum_{i=1}^{n}{\left(x_{i}-\mu_{x}\right)}^{2}{\left(y_{i}-\mu_{y}\right)}^{2}}}$$

where $\mu_{x}$ and $\mu_{y}$ mean the average of the variable *x* and *y*, respectively. The variable, including the level of bleeding classification using segmentation and non-segmentation are estimated as the correlation coefficient among the ground-truth dataset. The formulas return a value between −1 and 1. A correlation coefficient of 1 indicates a strong positive relationship. Otherwise, the correlation coefficient of zero means no relationship at all. The comparison of the correlation coefficient with the segmentation model and without segmentation model is shown in Table 4. The result shows that the system with the eliminating cutting loop fits better with the ground truth. The segmentation model can significantly optimize the ranking of bleeding level classification.

## 4. Conclusions

With the aim of assessing the ranking of bleeding level, the automated ranking of the bleeding level classification system for TURP surgery is proposed in this work. To avoid being disturbed by the red light easily, yielding from the surgical cutting loop during the ranking of bleeding level classification, the ResUNet model was utilized to eliminate the cutting loop. The experiment indicates the correlation value would be more fit the manual label by sufficient experienced urologists, while the segmentation model was implemented. Particularly, the four state-of-art classification models were utilized to assess the ranking of bleeding level. Considering the slight error allowable from the urologists, the revised accuracy of the four classification models can achieve 90, 89, 90, and 91%, respectively. More generally, the result demonstrates that the proposed methods have the ability to classify the ranking of bleeding level accurately and efficiently reduce the burden of urologists. We believe that our research has considerable potential because this method can assist in the performance assessment for surgical trainees of TURP. On the other hand, a surgical safety monitoring system can also be developed in the future based on this method to warn the surgeon when severe bleeding occurs during the operation to protect the safety of patients.

## Figures and Tables

**Table 1:** Qualitative comparison of the four machine learning models for evaluating the levels of blood loss in the validation stage.

Model	Scatter Plot	Confusion Matrix
KNN	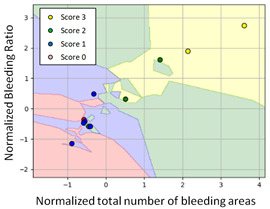	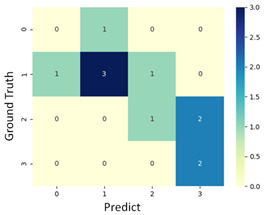
Naïve Bayes	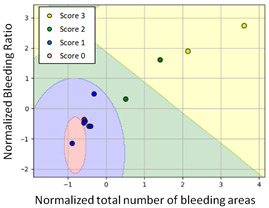	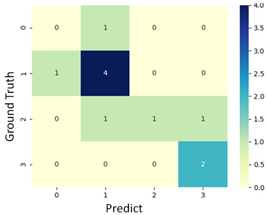
Random Forest	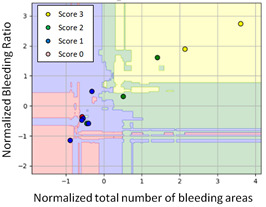	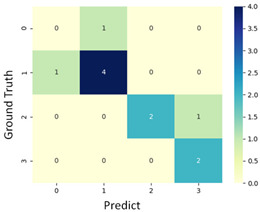
SVM	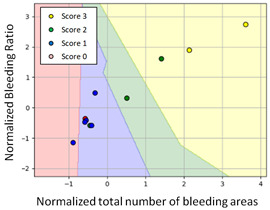	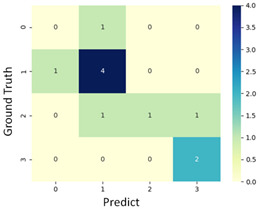

**Table 2:** Qualitative comparison of the four machine learning models for evaluating the levels of blood loss in the testing stage.

Model	Scatter Plot	Confusion Matrix	Revised Confusion Matrix
KNN	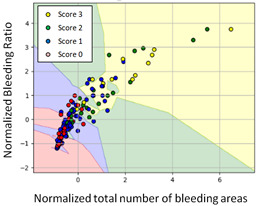	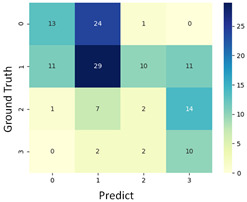	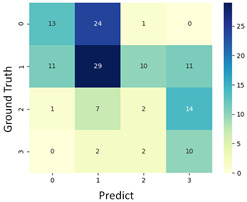
Naïve Bayes	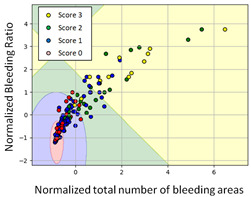	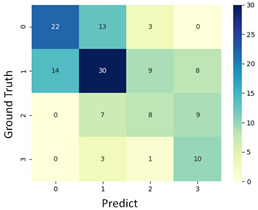	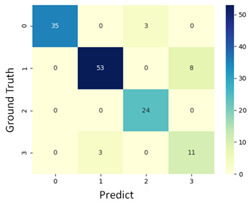
Random Forest	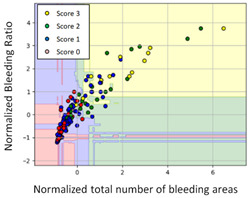	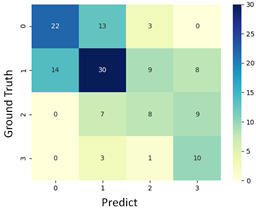	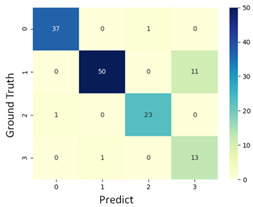
SVM	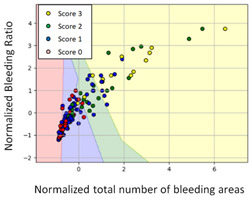	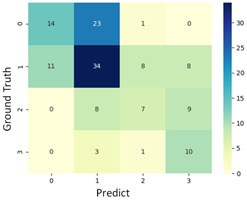	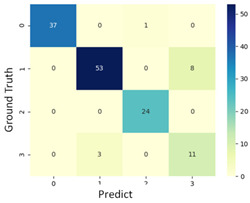

**Table 3:** The comparison of the four machine learning classifiers results between before and after being revised.

Model	Before Being Revised			Revised		
	Accuracy	Precision	Recall	Accuracy	Precision	Recall
KNN	0.39	0.35	0.40	0.90	0.85	0.89
Naïve Bayes	0.51	0.48	0.53	0.89	0.85	0.90
Random Forest	0.47	0.50	0.47	0.90	0.86	0.92
SVM	0.47	0.46	0.48	0.91	0.87	0.91

**Table 4:** The comparison of the correlation coefficient with the segmentation model and without segmentation model.

	With Segmentation Model	Without Segmentation Model
KNN	0.589	0.583
Naive Bayes	0.619	0.605
Random Forest	0.620	0.617
SVM	0.601	0.596

## Data Availability

The data are not publicly available.
